# Supplementary material for: DNA methylome regulates virulence and metabolism in Pseudomonas syringae
Source: eLife. 2025 Feb 24;13:RP96290. doi: 10.7554/eLife.96290 (PMC11850005; doi:10.7554/eLife.96290)
Supplement: Supplementary file 1. [file elife-96290-supp1.docx]

**Table S1** Restriction-modification systems predicted in *P. syringae*

| Strain | Type | Gene | Name | Predicted  Rec Seq | Coordinates |
| --- | --- | --- | --- | --- | --- |
| Psph | I | R | PSPPH_0104 | - | 116979-119966 |
| Psph | I | S | PSPPH_0106 | - | 121882-123228 |
| Psph | I | M | PSPPH_0107 | - | 123215-125620 |
| Psph | II | M | PSPPH_1883 | - | 2183856-2184650 |
| Psph | II | M | PSPPH_1894 | - | 2194525-2195319 |
| Psph | II | M | PSPPH_4982 | - | 5652396-5653190 |
| Pst | I | M | PSPTO_0005 |  | 7092-8798 |
| Pst | I | S | PSPTO_0006 |  | 8861-10168 |
| Pst | I | R | PSPTO_0008 |  | 11067-14366 |
| Pst | I | M | PSPTO_1086 |  | 1191539-1193269 |
| Pst | I | S | PSPTO_1087 |  | 1193259-1194527 |
| Pst | I | R | PSPTO_1089 |  | 1195573-1198695 |
| Pst | II | M | PSPTO_2386 |  | 2635219-2636106 |
| Pst | II | M | PSPTO_3388 |  | 3830042-3830836 |
| Pst | II | M | PSPTO_3427 |  | 3862800-3864584 |
| Pss | II | M | Psyr_1433 |  | 1621535-1622950 |
| Pss | II | M | Psyr_2825 |  | 3399004-3400125 |
| Pss | II | M | Psyr_2828 |  | 3401768-3403822 |
| Pss | II | M | Psyr_2843 |  | 3415442-3417574 |

I: type I RM modification system;

II: type II RM modification system;

M: methylase;

S: specificity;

R: restriction;
